# Rectal cancer treatment and outcome in the elderly: an audit based on the Swedish rectal cancer registry 1995–2004

**DOI:** 10.1186/1471-2407-9-68

**Published:** 2009-02-26

**Authors:** Bärbel Jung, Lars Påhlman, Robert Johansson, Erik Nilsson

**Affiliations:** 1 Department of Surgery and Perioperative Sciences, University of Umeå, Sweden; 2 Department of Surgery, Visby Hospital, Visby, Sweden; 3 Department of Surgery, Academic Hospital, Uppsala, Sweden; 4 Oncology Centre, Umeå University Hospital, Umeå, Sweden

## Abstract

**Background:**

Limited information is available regarding the effect of age on choice of surgical and oncological treatment for rectal cancer. The objective of this study was to assess the influence of age on treatment and outcome of rectal cancer.

**Methods:**

We utilized data in the Swedish Rectal Cancer Registry (SRCR) from patients treated for rectal cancer in Sweden in 1995–2004.

**Results:**

A total of 15,104 patients with rectal cancer were identified, 42.4% of whom were 75 years or older. Patients ≥75 years were less likely to have distant metastases than younger patients (14.8% vs. 17.8%, *P *< 0.001), and underwent abdominal tumor resection less frequently (68.5% vs. 84.4%, *P *< 0.001). Of 11,725 patients with abdominal tumor resection (anterior resection [AR], abdominoperineal excision [APE], and Hartmann's procedure [HA]), 37.4% were ≥75 years. Curative surgery was registered for 85.0% of patients ≥ 75 years and for 83.9% of patients < 75 years, *P *= 0.11. Choice of abdominal operation differed significantly between the two age groups for both curative and non-curative surgery, The frequency of APE was similar in both age groups (29.5% vs. 28.6%), but patients ≥75 years were more likely to have HA (16.9% vs. 4.9%) and less likely to have preoperative radiotherapy (34.3vs. 67.2%, *P *< 0.001). The relative survival rate at five years for all patients treated with curative intent was 73% (70–75%) for patients ≥75 years and 78% (77–79%) for patients < 75 years of age. Local recurrence rate was 9% (8–11%) for older and 8% (7–9%) for younger patients.

**Conclusion:**

Treatment of rectal cancer is influenced by patient's age. Future studies should include younger and older patients alike to reveal whether or not age-related differences are purposive. Local recurrence following surgery for low tumors and quality of life aspects deserve particular attention.

## Background

Rectal cancer predominantly affects persons over the age of 50. The annual number of rectal cancer cases diagnosed in Sweden has increased over the last twenty years, mainly due to the increasing age of the Swedish population, a trend that is expected to continue [1,2]. Previous studies indicate that there is less inclination to perform surgery and adjuvant oncological treatment in elderly patients [3-5]. There is, however, mounting evidence that fit, elderly patients may benefit from surgery and adjuvant oncological treatment in much the same way as younger patients [6-8].

Since it began in 1995, the Swedish Rectal Cancer Registry (SRCR) has prospectively registered nearly 100% of patients diagnosed with rectal cancer in Sweden, along with data describing choice of surgical strategy, postoperative morbidity and mortality, neoadjuvant treatment, and long-term survival[9]. The degree of coverage in the SRCR is 98–100% of patients with an adenocarcinoma of the rectum.

The aim of this audit was to investigate the influence in Sweden of age on choice of surgical strategy, use of preoperative radiotherapy, and outcome following abdominal surgery to treat rectal cancer.

## Methods

All patients in the SRCR diagnosed from 1995 through 2004 were divided into two groups: ≥75 years and < 75 years of age at diagnosis. Abdominal tumor resection was defined as anterior resection (AR), abdominoperineal excision (APE), and Hartmann's procedure (HA). Cohorts used and outcomes (parentheses) analyzed are summarized below.

1995–2004: Patients with rectal cancer diagnosis, *N *= 15,104 (distant metastases [yes/no]). Abdominal tumor resection, *N *= 11,725 (tumor stage). Curative/noncurative surgery known, *N *= 11,510. Curative surgery *N *= 9,705 (tumor location, preoperative radiotherapy, reoperation at 30 days, relative survival 90 days following surgery, and relative 5-year survival).

Curative surgery was defined as a local radical procedure (R0) with tumor-negative resection margins in patients with no sign of distant metastases, according to UICC.

The study was approved by the Ethics Committee of the University of Umeå, Sweden (05-097M).

### Statistics

The chi-square test was used to test differences between proportions. Two-tailed *P *values < 0.05 were considered significant. SPSS v 13.0 (SPSS Inc, Chicago Illinois) software was used for statistical analyses. The statistical program Relsurv version 1.0 [10] was utilised for analysis of relative survival, using data from the SRCR matched with data from The National Board of Health and Welfare's vital statistics [1,2]. Local recurrence was calculated according to Kaplan-Meier with censoring of patients deceased before 31 December 2001 or with rectal cancer diagnosis after December 2001. This time was chosen as end point because all patients undergoing surgery for rectal cancer with curative intent are checked for local recurrence five years after surgery by Swedish Regional Oncologic Centres, and patients treated after December 2001 had not yet completed this control.

Data are presented as mean (95% confidence interval).

## Results

### Tumor stage versus age and treatment

Between 1 January 1995 and 31 December 2004, 15,104 patients diagnosed with rectal cancer were identified in the SRCR. Of these, 42.4% were ≥75 years of age at diagnosis. The median age of patients ≥75 years was 80 years, and the median age was 65 years for those younger than 75 years. There was a lower proportion of men in the older group (54.1%) compared with the younger group (59.5%), *P *< 0.001. Among 6,407 patients ≥75 years (of total 15,104), 946 (14.8%) had distant metastases at diagnosis compared with 1,550 of 8,697 patients (17.8%) < 75 years of age, *P *< 0.001. Altogether, 11,725 of 15,104 patients (77.6%) had an abdominal tumor resection; 4,388 of 6,407 patients ≥75 years (68.5%), and 7,337 of 8,697 patients < 75 years (84.4%), *P *< 0.001.

Tumor stage distribution according to age group among those who underwent abdominal surgery is shown in Table 1. Information on tumor stage was missing for 170 patients (1.5%); 74 of those who underwent AR (1.1%), 59 of those who underwent APE (1.7%), and 37 of those who underwent HA (2.4%). Older patients were less likely to have Stage IV disease than younger patients (10.3% vs. 13.4%, *P *< 0.001). Patients who underwent HA were more likely to have Stage IV disease (27.9%) compared with patients who underwent AR (9.8%) or APE (10.3%). A total of 84.3% of patients were assessed by the surgeon as having an R0 resection: 85.0% in the older group and 83.9% in the younger group, *P *= 0.11. Two hundred and fifteen patients were excluded from further analysis due to incomplete information concerning curative and noncurative surgery. Thus, 11,510 patients were eligible for evaluation of perioperative and postoperative data (reoperation rate at 30 days, relative survival at 90 days following surgery and relative survival at 5 years).

**Table 1:** Type of operation and tumor stage versus age of patients with abdominal tumor resection (*N *= 11,725)

Operation	Tumor stage	< 75 years of age	≥ 75 years of age	*P *value
AR		*N *= 4,514	*N *= 2,235	0.001
	Stage I-III	3,980 (88.2)	2,035 (91.1)	
	Stage IV	475 (10.5)	185 (8.3)	
	Stage not known	59 (1.4)	15 (0.6)	
APE		*N *= 2181	*N *= 1,277	0.001
	Stage I-III	1,887 (86.5)	1,155 (90.4)	
	Stage IV	246 (11.3)	111 (8.7)	
	Stage not known	48 (2.2)	11 (0.9)	
HA		*N *= 642	*N *= 876	< 0.001
	Stage I-III	362 (56.4)	696 (79.5)	
	Stage IV	266 (41.4)	157 (17.9)	
	Stage not known	14 (2.2)	23 (2.6)	
Abdominal surgery		*N *= 7,337	*N *= 4,388	< 0.001
	Stage I-III	6,229 (84.9)	3,886 (88.6)	
	Stage IV	987 (13.4)	453 (10.3)	
	Stage not known	121 (1.7)	49 (1.1)	

Values are absolute numbers with percentages given in brackets. Chi-squared test used for analysis of difference between age groups. AR, anterior resection. APE, abdominoperineal excision. HA, Hartmann's procedure.

As shown in Table 2, the surgical strategy for abdominal resection varied with patient age. The percentage of APE was similar in the two age groups both for curative and noncurative operations. AR was less commonly performed in elderly patients, whereas the reverse was true for HA. These age-related differences were more pronounced for curative surgery, but they were also significant for noncurative surgery.

**Table 2:** Choice of operation for curatively and noncuratively treated patients (*N *= 11,510).

	Operation	< 75 years	≥ 75 years	All patients
Curative surgery		*N *= 6,042	*N *= 3,663	*N *= 9,705
	AR	3,965 (65.6)	1,998 (54.5)	5,963 (61.4)
	APE	1,783 (29.5)	1,047 (28.6)	2,830 (29.2)
	HA	294 (4.9)	618 (16.9)	912 (9.4)
Noncurative surgery		*N *= 1,160	*N *= 645	*N *= 1,805
	AR	485 (41.8)	214 (33.2)	699 (38.7)
	APE	350 (30.2)	208 (32.2)	558 (30.9)
	HA	325 (28.0)	223 (34.6)	548 (30.4)
Total		7,202	4,308	11,510

Values are absolute numbers with percentages given in brackets.

*P *< 0.001 for both curative and noncurative surgery. Chi-squar test was used for analysis of difference between age groups. AR, anterior resection. APE, abdominoperineal excision. HA, Hartmann's procedure.

Table 3 illustrates the proportion of low tumors (tumor 0–6 cm from the anal verge) for each surgical procedure in the two age groups. In the majority of cases for both age groups, APE was chosen as surgical strategy for low tumors (0–6 cm from anal verge), AR was used slightly but significantly less for low tumors among older patients, 10.1% versus 12.9% for younger patients, *P *< 0.003. Table 4 illustrates the use of preoperative radiotherapy for patients treated with curative intent in the two age groups.

**Table 3:** Low tumors (0–6 cm from anal verge; N = 3445) versus type of operation and age in curatively treated patients (N = 9705).

	< 75 years of age Low tumors (N = 2145)/curatively treated (N = 6042)	≥ 75 years of age Low tumors (N = 1114)/Curatively treated (N = 3663)	P-value
AR	510/3965	202/1998	0.003
APE	1560/1783	912/1047	0.86
HA	75/294	186/618	0.19

Values are absolute numbers of patients with low tumor for each surgical procedure vs. age group. Chi-square test used for statistical analysis. AR, anterior resection. APE, abdominoperineal excision. HA, Hartmann's procedure.

**Table 4:** Use of preoperative radiotherapy versus surgical procedure and age in curatively treated patients (N = 9705).

Curative surgery	< 75 years of age *N *= 6,042	≥ 75 years of age *N *= 3,663	*P*-value
Preoperative radiotherapy	4,061 (67.2)	1,255 (34.3)	P < 0.001
AR	2,436/3,965 (61.4)	635/1,998 (31.8)	P < 0.001
APE	1,457/1,783 (81.7)	495/1,047 (47.3)	P < 0.001
HA	168/294 (57.1)	125/618 (20.2)	P < 0.001

Values are absolute numbers of patients receiving radiotherapy for each surgical procedure versus age group. Percentage given in brackets. Chi-square test used for analysis of difference between age groups. AR, anterior resection. APE, abdominoperineal excision. HA, Hartmann's procedure.

As can be seen, older patients received preoperative radiotherapy less frequently than younger patients regardless of the surgical technique used, overall 34.3% versus 67.2%, for patients having curative surgery *P *< 0.001.

Reoperation within 30 days of curative surgery was performed in 340 of 3,663 patients (9.3%) ≥75 years and in 642 of 6,042 patients (10.3%) < 75 years, *P *= 0.036. The reoperation rate for each procedure did not, however, differ significantly between the age groups.

### Relative survival

Relative survival 90 days postoperatively was slightly but significantly lower for older patients compared with younger patients following curative abdominal tumor resection: AR 96% (95–97%), mean (95% CI) vs. 99% (98.6–99.4%); APE 97% (96–98%) vs. 99% (98.5–99.5%); and HA 94% (92–96%) vs. 98%(96.7–100%). For all patients ≥75 years, relative survival 90 days following surgery was 96% (95–97%) compared with 99% (98.7–99.3%) for younger patients.

For all 15,104 patients registered in SRCR 1995–2004, the relative five-year survival was 62% (61–63%) for patients < 75 years vs. 52% (50–54%) for patients ≥75 years.

**Figure 1:**
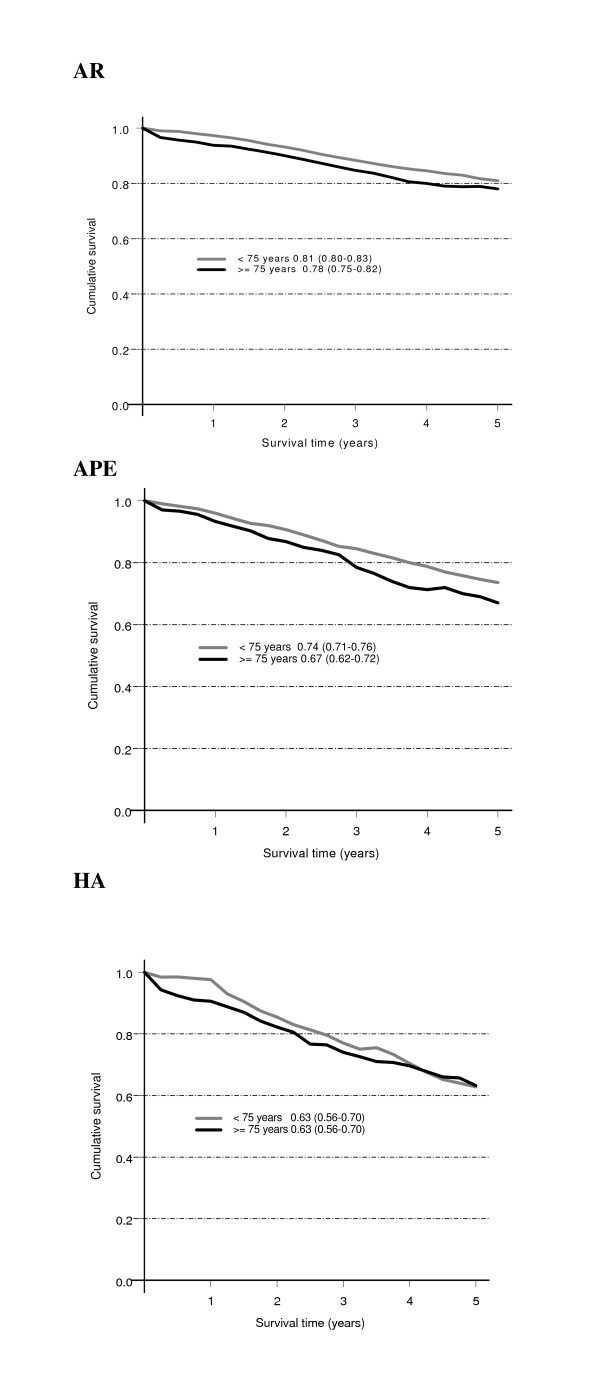
**Relative five-year survival rate (95% confidence interval) for patients curatively treated with abdominal surgery for rectal cancer 1995–2004**. *N *= 9,705 (of 11,510 curative and noncurative operations). AR, anterior resection. APE, abdominoperineal excision. HA, Hartmann's procedure.

For all patients with abdominal tumor resection the relative five-year survival (95% confidence interval) was 68% (67–69%) for patients < 75 years vs. 64% (61–66%) for patients ≥75 years. Among patients who underwent abdominal tumor resection with curative intent from 1995 through 2004 (*N *= 9,705), relative five-year survival was significantly lower for those ≥75 years compared with patients < 75 years, 73% (70–75%) vs. 78% (77–79%). For the specific abdominal operations, there was a tendency towards lower relative survival for APR in the elderly (although with overlapping 95% confidence limits), (Figure 1).

### Local recurrence

**Figure 2:**
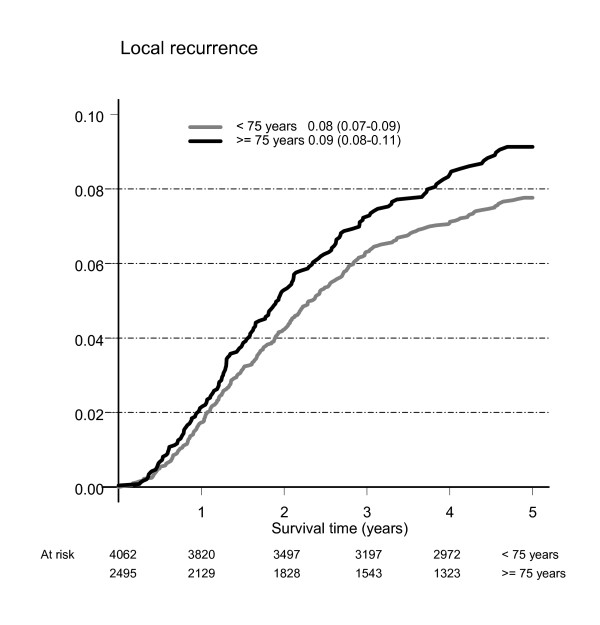
**Local recurrence rates shown as relative risk of local recurrence relative to population at risk 1995–2001 (curatively treated for rectal cancer)**. Mean (95%CI).

Local recurrence rate was 8% (7–9%) for patients < 75 years and 9% (8–11%) for those ≥ 75 years with no significant difference between the age groups, see Figure 2. There was no significant difference in this respect between the two age groups for any of the three abdominal tumour resection procedures, see Table 5.

**Table 5:** Local recurrrence rate

	< 75 years	≥ 75 years
AR	7% (6–8%)	8% (7–10%)
APE	9% (8–11%)	10% (7–12%)
HA	12% (7–18%)	12% (8–16%)

Calculation based on 6557 curative operations 1995–2001. Values given as percentages with 95% confidence interval in brackets. AR, anterior resection, APE, abdominoperineal excision, HA, Hartmann's procedure.

## Discussion

The main findings in this study can be summarized as follows: Among patients 75 years or older, distant metastases were diagnosed less frequently than in younger patients. Older patients underwent abdominal surgery less frequently but they had more HA:s than younger patients. Preoperative radiotherapy was used for 34% of patients ≥75 years, compared with 67% of younger patients. Older patients had lower relative survival 90 days postoperatively and lower relative five-year survival among all patients, among patients with abdominal tumor resection, and among patients with curative operation. Local recurrence rate did not, however, differ significantly between the two age groups.

The SRCR covers nearly 100% of rectal cancer patients in Sweden, providing an adequate description of the management of rectal cancer in a defined population[9]. It has the possibility to use person-numbers, unique for each resident in Sweden. The register is validated and the quality of data has been shown to be acceptable. This makes descriptive data from this register of interest when planning studies validating future treatment programs for rectal cancer. The SRCR is nationwide and therefore the data input is limited to ensure acceptable registration compliance. American Association of Anesthesiologists (ASA) classification has been added to the registry in 2007. This information could have been helpful in trying to understand some of the results of our study, since older patients are expected to have more comorbidity, as shown in previous studies [11-13].

Local recurrence rate in our audit was calculated using a cohort different from the main cohort. The reason for this is the method of gathering information about local recurrence from colorectal units to Regional Oncologic Centres yearly, asking for local recurrence data for every patient five years post year of surgery.

The choice of surgical strategy for tumors more than 6 cm from the anal verge differed substantially between the older and younger age groups. HA was chosen more often for older patients with less advanced disease, which does not concur with a previous report, where sphincter saving surgery was used in the majority of patients in both age groups[8]. This is the most likely explanation for the tendency towards a better five-year relative survival rate in older patients compared with younger patients treated with HA in our audit. The preference for HA in elderly patients in our audit, as well as in a study from Norway[14], could be due to concern about the functional result (risk for fecal incontinence) after restorative surgery in older patients. There is some evidence that the functional result after AR is acceptable in older patients[15], and chronological age alone should not exclude patients from restorative surgery for rectal cancer. However, available studies comparing quality of life in patients treated for rectal cancer with a permanent stoma (HA) and restorative surgery (AR) do not demonstrate a significant difference between the two alternatives [16,17]. Further prospective studies in this aspect are important, since the short-term and long-term survival did not differ significantly between patients in the AR and HA groups in our audit.

A slightly lower relative survival at 90 days for elderly patients highlights the physiological age limit for major abdominal surgery. Although the reoperation rate differed very little between the age groups, one may anticipate that reoperation imposes a greater risk in elderly patients compared with younger patients. Older patients who underwent abdominal tumor resection had a less favorable relative five-year survival than younger patients (64% vs. 68%), in spite of less advanced tumor stage (10.3 vs 13.4 stage IV). The five-year relative survival was also lower for older patients than for younger patients treated with curative intent. It is of special interest that preoperative radiotherapy prior to APE was used in 47% of patients ≥ 75 years, compared with 82% in younger patients. However, no significant difference in the five-year local recurrence rate after APE was seen. This indicates that preoperative radiotherapy alone cannot entirely explain the slightly worse five-year relative survival in older patients.

The total mesorectal excision technique (TME) [18-22] was introduced in Sweden during the latter half of the 1990s after initial studies[23]. A similar introduction took place during the same period in Norway[17,24]. The impact of the TME technique in reducing the local recurrence rate is evident from population-based educational programs[22,24]. There is ongoing discussion regarding the need for improvement in APE surgical techniques for better local control in order to improve outcome and possibly to reduce the need for preoperative radiotherapy[9,25]. Population-based studies with prospective detailed registration of surgical technique may shed light on this important issue.

The extent to which preoperative radiotherapy should be used in rectal cancer treatment is controversial. Preoperative radiotherapy is known to improve local control but it also increases the probability of fecal incontinence, sexual dysfunction, and late hospital admission [26-31]. Negative side effects have been more pronounced in older patients, as was shown in the Stockholm I and II trials[32,33]. This is probably one factor explaining the low rate of preoperative radiotherapy in the older age group in our audit. However, it should be noted that modern radiotherapy has a higher tolerability than techniques used in previous trials. Further studies, preferably with a cost-benefit approach, concerning preoperative radiotherapy are needed.

## Conclusion

A higher threshold for resectional surgery was seen in elderly patients, who were less likely to have stage IV disease at operation. This age-dependent difference in tumor stage was identified in all operations. It was most pronounced for HA, frequently used in elderly patients. Five-year relative survival was lower for all elderly patients and for elderly patients treated with curative intent, whereas local recurrence rate was comparable in the two age groups. In order to improve outcomes for patients with rectal cancer both population-based studies and trials comprising older and younger patients, with emphasis on physiological and quality-of-life aspects, are needed.

## Competing interests

The authors declare that they have no competing interests.

## Authors' contributions

BJ, EN, and LP participated in the design of the study. RJ processed data from SRCR and made statistical calculations. BJ drafted the manuscript. All authors participated in the revision of the manuscript and approved the final version of the manuscript.

## Pre-publication history

The pre-publication history for this paper can be accessed here:

http://www.biomedcentral.com/1471-2407/9/68/prepub
